# Maternal smoking during infancy increases the risk of allergic diseases in children: a nationwide longitudinal survey in Japan

**DOI:** 10.1186/s13223-025-00952-9

**Published:** 2025-01-16

**Authors:** Kenji Shigehara, Naomi Matsumoto, Mitsuru Tsuge, Kazuhiro Uda, Yukie Saito, Masato Yashiro, Takashi Yorifuji, Masanori Ikeda, Hirokazu Tsukahara

**Affiliations:** 1 Department of Pediatrics, Okayama University Graduate School of Medicine, Dentistry, and Pharmaceutical Sciences, Okayama, Japan; 2 Department of Pediatrics, Okayama University Hospital, Okayama, Japan; 3 Department of Epidemiology, Okayama University Graduate School of Medicine, Dentistry, and Pharmaceutical Sciences, Okayama, Japan; 4 Department of Pediatric Acute Diseases, Okayama University Graduate School of Medicine, Dentistry, and Pharmaceutical Sciences, 2-5-1 Shikata-cho, Kita-ku, Okayama, 700-8558 Japan

**Keywords:** Allergic rhinitis, Bronchial asthma, Atopic dermatitis, National cohort study, Passive smoking

## Abstract

**Background:**

The incidence of allergic diseases has been increasing in Japan. In particular, a serious decline in the age of onset of allergic rhinitis has been observed. Passive smoking from parental smoking has a significant impact on children’s health; however, it is difficult to restrict smoking in the home. While various studies have previously reported on the relationship between passive smoking and the development of allergic diseases in children. However, there have been no reports on passive smoking and allergic diseases on a national scale.

**Methods:**

Using Japanese national longitudinal survey data (*n* = 38,444) for newborns born between May 10 and 24, 2010, we assessed parental smoking habits when their children were 6 months old and investigated the association with the development of allergic diseases until the age of 5.5 years. The risk ratios and 95% confidence intervals for the development of different allergic diseases were analyzed after adjusting for potential confounders using Poisson regression with a robust error variance.

**Results:**

The risk ratio for developing allergic rhinitis/allergic conjunctivitis (AR/AC) in children was significantly higher in the maternal smoking groups ( ≦ 10 cigarettes/day; RR 1.15, 95% CI 1.02–1.30; ≧11 cigarettes/day; RR 1.16, 95% CI 0.93–1.44). Furthermore, associations were found between the maternal smoking group in the presence of paternal smoking and the risk of developing bronchial asthma ( ≦ 10, RR 1.33 95% CI 1.17–1.52; ≧11, RR 1.71 95% CI 1.38–2.1), food allergy ( ≦ 10, RR 1.36 95% CI 1.12–1.63; ≧11, RR 1.25 95% CI 0.84–1.86), atopic dermatitis ( ≦ 10, RR 1.42 95% CI 1.22–1.66; ≧11, RR 1.6 95% CI 1.2–2.13), and AR/AC ( ≦ 10, RR 1.21 95% CI 1.07–1.36; ≧11, RR 1.35 95% CI 1.09–1.67).

**Conclusions:**

Maternal smoking during infancy increases the risk of developing AR/AC in children. Considering paternal smoking, maternal smoking further increased the risk of developing allergic diseases in children, suggesting that reducing parental smoking at home may reduce the risk of developing allergic diseases in children.

**Supplementary Information:**

The online version contains supplementary material available at 10.1186/s13223-025-00952-9.

## Background

Passive smoking is defined as the inhalation of either the smoke exhaled by a smoker, or the sidestream smoke exhaled when a cigarette is overheated [1]. Passive smoking has been well-established to have a significant impact on health, and is known to cause a variety of diseases, including lung cancer, ischemic heart disease, stroke, chronic obstructive pulmonary disease, and lifestyle-related diseases in adults [1, 2]. Nonsmokers are highly sensitive to tobacco smoke, and even low-level passive smoking exposure can result in serious health problems [3]. Parental smoking is a common cause of passive smoking in children, and is associated with the onset of a variety of diseases, including sudden infant death syndrome and bronchial asthma (BA) [4, 5]. The World Health Organization (WHO) legislation on the prevention of passive smoking is aligned with Article 8 of the Framework Convention on Tobacco Control [6], which prohibits smoking in all indoor work and public places. This has led to the separation of smoking and smoking areas in public facilities worldwide; however, such a separation of smoking and non-smoking areas at home is difficult, while passive smoking from parents still exerts a significant impact on children’s health.

The number of pediatric patients with allergic diseases is increasing worldwide, including in Japan [7], and environmental factors have been suggested to influence the onset of these diseases. Many previous studies have already reported an association between passive smoking and the development of allergic diseases in children. Perinatal exposure to passive smoke has also been shown to increase the risk of developing food allergy (FA) in childhood [8], while maternal smoking during pregnancy is associated with the development of atopic dermatitis (AD) in infants [9–11]. Additionally, the onset of BA in children is associated with passive smoking from the parents [12, 13], while passive smoking exposure in cars is associated with the onset and worsening of allergic rhinitis (AR) in children [14]. However, most previous studies on this topic have been retrospective or cohort studies conducted in limited regions, and no cohort study with consistent long-term follow-up on a nationwide scale has yet been reported in Japan.

In the present study, we investigated the risk of developing allergic diseases in children exposed to passive smoke from their parents at 6 months of age, using data from a large national longitudinal cohort study in Japan.

## Methods

### Study design, setting, and participants

The Longitudinal Survey of Babies in the 21st century is a national representative longitudinal survey conducted by Japan’s Ministry of Health, Labour and Welfare. This survey follows the same individuals over a long period, in order to obtain basic information for administrative measures to solve problems such as the declining birthrate. This study followed babies born between May 10 and May 24, 2010. Baseline questionnaires were sent to all families when the surveyed infants were 6 months old. Of the 43,767 questionnaires delivered, 38,444 were completed and returned, representing an 87.8% response rate. Follow-up questionnaires were sent to all participants who initially responded at 1-year intervals between 18 months and 5.5 years of age (at 18, 30, 42, 54, and 66 months). Birth record data from Japanese vital statistics (weight; gestational age; singleton, twin, or other multiple birth; sex; birth number; maternal parity; parental age) were documented for each child included in the study. Furthermore, information on daycare attendance, parents’ educational level, residential area (ward, city, town, or village; categorical), and pet ownership were obtained using questionnaires from The Longitudinal Survey of Babies in the 21st century, a national representative longitudinal survey.

### Exposure and outcome definition

This study assessed whether parents smoked when their children were 6 months old, based on their responses to the questionnaire. The number of cigarettes that both parents smoked was recorded on the questionnaire. Because heat-not-burn tobacco and e-cigarettes were not sold in Japan until 2015, this study focused solely on cigarette smoking.

BA, FA, AD, or AR/ allergic conjunctivitis (AC) were selected as the allergic diseases of interest. The primary outcome of this study was whether a child received at least one diagnosis of any of the allergic diseases in the following age intervals from 1.5 to 5.5 years: 6–18 months, 18–30 months, 30–42 months, 42–54 months, and 54–66 months. Data regarding diagnosis for each allergic disease was obtained from the answers to a question asking parents whether they had undergone medical evaluation for the relevant disease in the past year. According to the interview format, AR and AC were grouped together into one category.

### Statistical analysis

First, demographic characteristics were distinguished by the maternal smoking or not categories. Some participants were lost to follow-up at the second or later surveys. To evaluate the impact of loss to follow-up, we examined which children in each category tended to be lost at each survey, and further compared the baseline characteristics between children included in the analysis and children lost to follow-up at each survey.

The potential confounders, including child and parental factors, as well as residential information, were selected based on previous studies or prior knowledge of the association between maternal smoking and allergic diseases [15, 16]. Child factors included sex (dichotomous), singleton or multiple births (dichotomous), term or preterm birth (< 37 weeks of gestation; dichotomous), birth weight (< 2500 g; dichotomous), birth number (first-born, second-born, and third-born or higher), and daycare attendance (dichotomous). Parental factors included maternal age at delivery (< 25, 25–29, 30–34, and ≥ 35 years; categorical), maternal educational level (categorical), and paternal educational level (categorical). Exposure information included maternal smoking habits (non-smoker; smoker, ≤ 10 cigarettes per day; and smoker, > 10 cigarettes per day; categorical) and paternal smoking habits (non-smoker; smoker, ≤ 10 cigarettes per day; and smoker, > 10 cigarettes per day; categorical). Residential information included the type of residential area where the participant was born (ward, city, town, or village; categorical). In the second survey, respondents were asked who usually took care of the children, and we assumed that children reported as being taken care of by nursery school teachers were attending a daycare center. Maternal and paternal educational levels were used as an indicator of socio-economic status, with information obtained from the second survey (age 18 months). Parents were classified into 4 educational categories, as follows: university (4 years) or higher, junior college, high school, and junior high school or others. Residential information was obtained from the national census conducted in 2010. The cat and dog ownership statuses were ascertained in the fourth survey.

Risk ratios (RR) and 95% confidence intervals (CI) for the development of BA, FA, AD, and AR/AC in children were analyzed based on the maternal smoking habits at 6 months of age. Potential confounders were adjusted using Poisson regression with a robust error distribution. Cases with missing data were excluded, and analyses were performed using only cases with complete data. A sensitivity analysis was performed by stratifying maternal and paternal smoking status. We evaluated effect modification by stratifying the analysis by paternal smoking status. Additionally, we assessed the additive interaction by calculating the relative excess risk due to interaction (RERI), and the multiplicative interaction by including a product term of maternal and paternal smoking in the model. All analyses were adjusted using the covariates indicated above. Stata SE version 17 statistical software (Stata Corp., College Station, TX, USA) was used for all analyses. This study was approved by the Institutional Review Board of the Okayama University Graduate School of Medicine, Dentistry, and Pharmaceutical Sciences (No. 1506-073).

## Results

A total of 38,444 children with available information on maternal smoking status at 6 months of age were analyzed. The demographic characteristics of the 2,687 cases in the maternal smoking group and 35,757 cases in the non-maternal smoking group at 6 months of age are shown (Table 1**)**. There were no differences between the two groups in terms of sex, single or multiple births, gestational age, singleton or multiple births, nursery school/kindergarten attendance status, or residential area. The most common age group for smoking mothers was 25 to 29 years old, while that for non-smoking mothers was 30 to 34 years old. The most common educational attainment for smoking mothers was high school, while that for non-smoking mothers was junior college. The most common educational attainment for smoking fathers was high school, while the most common educational attainment for non-smoking fathers was university or higher. Therefore, parents’ educational attainment tended to be higher in the non-smoker group than in the smoker group. The rate of pet ownership in the smoking mother group was approximately twice as high as in the non-smoking mother group.

**Table 1:** Baseline characteristics of the parents and infants at 6 months of age

	Mother	
	Smoking (*n* = 2,687)	Non-smoking (*n* = 35,757)
**Gender, n (%)**		
Male	1,352 (50.3)	18,444 (51.6)
Female	1,335 (49.7)	17,313 (48.4)
**Singleton or multiple birth, n (%)**		
Singleton birth	2,635 (98.1)	35,086 (98.1)
Multiple birth	52 (1.9)	671 (1.9)
**Preterm birth, n (%)**		
22–36 weeks	169 (6.3)	1,922 (5.4)
37 weeks or more	2,518 (93.7)	33,828 (94.6)
**Birth weight, n (%)**		
< 2500	2,345 (87.3)	32,456 (90.8)
≥ 2500 g	342 (12.7)	3,294 (9.2)
**Birth order, n (%)**		
First	988 (36.8)	17,042 (47.7)
Second	978 (36.4)	13,347 (37.3)
Third or later	721 (26.8)	5,368 (15.0)
**Daycare, n (%)**		
Yes	634 (32.8)	8,594 (27.4)
No	1,297 (67.2)	22,739 (72.6)
**Mother age, n (%)**		
< 25	613 (22.8)	3,141 (8.8)
25–29	784 (29.2)	10,076 (28.2)
30–34	748 (27.8)	13,441 (37.6)
> 35	542 (20.2)	9,099 (25.4)
**Mother educational attainment, n (%)**		
University or higher	107 (5.6)	8,670 (27.7)
Junior college	473 (24.6)	13,190 (42.2)
High school	901 (46.7)	8,185 (26.2)
Junior high school or others	446 (23.1)	1,226 (3.9)
**Father educational attainment, n (%)**		
University or higher	252 (13.9)	14,111 (45.8)
Junior college	282 (15.6)	5,705 (18.5)
High school	851 (47.2)	9,167 (29.7)
Junior high school or others	420 (23.3)	1,835 (6.0)
**Residential area, n (%)**		
Wards	697 (25.9)	10,281 (28.8)
City	1,720 (64.0)	22,617 (63.2)
Town or villages	270 (10.1)	2,859 (8.0)
**Pet ownership (cat and/or dog), n (%)**		
Yes	344 (23.3)	3,373 (12.3)
No	1,133 (76.7)	23,952 (87.7)

Maternal and paternal educational levels were used as an indicator of socio-economic status, with information obtained at the second survey (18 months of age). Information on pet ownership was obtained at the fourth survey (42 months of age)

Of the 38,444 families that responded to the survey at 6 months of age, 11,973 had dropped out by the time of the survey at 5.5 years of age. Therefore, to evaluate the impact of loss to follow-up, we compared the baseline characteristics between children who were included in the analysis and children who were lost to follow-up at 5.5 years of age (6th survey). Loss to follow-up were more common among younger mothers and less educated parents, as well as among those whose parents both smoked (Supplemental Table).

We further analyzed the risk of developing each allergic disease in children until the age of 5.5 years, based on maternal smoking when the children were 6 months old (Table 2). In the non-smoking group, 5,686 (27.1%) developed AR/AC. In contrast, in the smoking group, 315 developed AR/AC, of whom 241 (34.2%) had mothers smoking less than 10 cigarettes per day and 74 (36.6%) had mothers smoking 11 or more cigarettes per day. The RR for developing AR/AC in children was significantly higher in the group of smoking mothers ( ≦ 10 cigarettes/day; RR 1.15, 95% CI 1.02–1.30). However, a dose-response relationship was not found for the number of cigarettes smoked by mothers (RR 1.16, 95% CI 0.93–1.44). The risk for developing FA, BA, or AD in children was no higher in the maternal smoking group.

**Table 2:** Relationship between maternal smoking during infancy and risk of developing allergic disease at 5.5 years of age

	Maternal smoking (cigarettes/day)	Adjusted model		
		n / N (%)	RR	95% CI
Allergic rhinitis/ Allergic conjunctivitis	0	5,686 / 20,943 (27.1)	1	-
	≦ 10	241 / 705 (34.2)	1.15	1.02–1.30
	≧ 11	74 / 202 (36.6)	1.16	0.93–1.44
Food allergy	0	2,669 / 20,344 (13.1)	1	-
	≦ 10	118 / 664 (17.8)	1.24	1.00-1.53
	≧ 11	27 / 182 (14.8)	0.84	0.50–1.41
Bronchial asthma	0	4,089 / 20,772 (19.7)	1	-
	≦ 10	208 / 713 (29.2)	1.14	0.95–1.37
	≧ 11	76 / 215 (35.3)	1.18	0.84–1.67
Atopic dermatitis	0	3,221 / 20,473 (15.7)	1	-
	≦ 10	154 / 688 (22.4)	1.14	0.95–1.37
	≧ 11	44 / 190 (23.2)	1.18	0.84–1.67

Abbreviations: N, number of children; RR, risk ratio; CI, confidence interval

Adjusted for child factors (sex, singleton or not, preterm birth and day care attendance), parental factors (maternal age at delivery, maternal educational attainment and paternal educational attainment) and residential area

In order to analyze the risk of developing each allergic disease in children due to parental smoking, we stratified maternal smoking habits according to paternal smoking habits (Table 3). The maternal smoking group in the presence of paternal smoking was associated with a significantly higher risk of developing AR/AC in children, regardless of the number of cigarettes the mother smoked per day (≤ 10, RR 1.21 95% CI 1.07–1.36; ≥11, RR 1.35 95% CI 1.09–1.67). Children with the maternal smoking group in the presence of paternal smoking had a significantly higher risk of developing FA, although the RR was not significantly higher when the mother smoked 11 cigarettes/day or more (RR 1.25, 95% CI 0.84–1.86). Children with maternal smoking group in the presence of paternal smoking had a significantly higher risk of developing BA or AD, regardless of the number of cigarettes the mother smoked per day (BA; ≤10, RR 1.33 95% CI 1.17–1.52; ≥11, RR 1.71 95% CI 1.38–2.1, AD; ≤10, RR 1.42 95% CI 1.22–1.66; ≥11, RR 1.60 95% CI 1.20–2.13). Among the maternal smoking group in the presence of paternal smoking, the RR for developing AR/AC, BA, and AD in children tended to increase in the group with mothers who smoked 11 or more cigarettes per day compared to the group whose mothers smoked 10 or fewer cigarettes per day. None of the multiplicative and additive interactions for maternal and paternal smoking showed significance.

**Table 3:** Relationship between maternal smoking during infancy and risk of developing allergic diseases stratified by paternal smoking at 5.5 years of age

	Maternal smoking (cigarettes/day)	Non-paternal smoking			Paternal smoking		
		n / N (%)	RR	95% CI	n / N (%)	RR	95%CI
Allergic rhinitis/ Allergic conjunctivitis	0	3,600 / 13,468 (26.7)	1	-	2,002 / 7,206 (27.8)	1	-
	≦ 10	30 / 82 (36.6)	1.37	1.03–1.82	200 / 595 (33.6)	1.21	1.07–1.36
	≧ 11	8 / 33 (24.2)	0.91	0.50–1.66	54 / 144 (37.5)	1.35	1.09–1.67
Food allergy	0	1,701 / 13,084 (13.0)	1	-	932 / 7,002 (13.3)	1	-
	≦ 10	9 / 74 (12.2)	0.94	0.51–1.73	101 / 560 (18.0)	1.36	1.12–1.63
	≧ 11	4 / 34 (11.8)	0.9	0.36–2.27	21 / 126 (16.7)	1.25	0.84–1.86
Bronchial asthma	0	2,417 / 13,283 (18.2)	1	-	1,587 / 7,216 (22.0)	1	-
	≦ 10	22 / 85 (25.9)	1.42	0.99–2.04	176 / 600 (29.3)	1.33	1.17–1.52
	≧ 11	8 / 36 (22.2)	1.22	0.66–2.25	57 / 152 (37.5)	1.71	1.38–2.1
Atopic dermatitis	0	2,001 / 13,156 (15.2)	1	-	1,180 / 7,060 (16.7)	1	-
	≦ 10	11 / 78 (14.1)	0.93	0.54–1.61	138 / 581 (23.8)	1.42	1.22–1.66
	≧ 11	6 / 35 (17.1)	1.13	0.54–2.34	35 / 131 (26.7)	1.6	1.2–2.13

Abbreviations: N, number of children; RR, risk ratio; CI, confidence interval

Adjusted for child factors (sex, singleton or not, preterm birth and day care attendance), parental factors (maternal age at delivery, maternal educational attainment and paternal educational attainment) and residential area

## Discussion

In this study, we investigated the risk of children developing allergic diseases until 5.5 years of age, depending on parental smoking status at 6 months of age, using data from a large-scale national longitudinal study in Japan. This study revealed that maternal smoking during infancy increased the risk of children developing AR/AC, and that the maternal smoking group in the presence of paternal smoking increased the risk of children developing AR/AC, FA, BA, and AD. To the best of our knowledge, this is the first study using large-scale national longitudinal data to analyze the risk of developing these four types of allergic diseases in children, and the first to assess the influence of passive smoking exposure from each parent.

This study showed that maternal smoking at 6 months of age significantly increased the child’s risk of developing AR/AC. Prior reports have shown that passive smoking from parents increases the risk of developing AR in children. Indeed, a study of 2,809 children aged 13 to 14 using the ISAAC (International Study of Asthma and Allergies in Childhood) questionnaire revealed that exposure to passive smoking from parents in cars was associated with increased risk of the development of seasonal AR in children [14]. Exposure to passive smoking at 1, 2, and 4 years of age has also been reported to significantly increase the incidence of childhood rhinitis [8]. A study of 1,315 Asian children reported that high serum cotinine concentrations were associated with both previous and current AR [17]. In a study of 68 children with AR caused by tick sensitization, the number of eosinophils in the nasal secretions was significantly higher in the group exposed to passive smoking than in the non-exposed group, regardless of the history of anti-allergy medication or sublingual immunotherapy [18]. Conversely, in a cross-sectional study of 671 children aged 2–7 years, no significant increase in the risk of developing AR was observed in children exposed to passive smoking from the parents [19]. In addition to AR, passive smoking may also increase the risk of developing AC [20]; however, no research reports have yet shown a clear association. Because the questionnaire used in our study did not distinguish between AR and AC, with these two conditions collated as AR/AC, we were unable to analyze the risk of developing AC alone. In our study, the child’s risk of developing AR/AC did not increase depending on the number of cigarettes smoked by the mother. However, previous studies have reported that increasing the number of cigarettes exposed to during passive smoking per day further increases the exacerbation of AR symptoms [21], and that passive smoking of 20 or more cigarettes per day increases the risk of developing AR compared to passive smoking of less than 20 cigarettes per day, or no exposure [22]. However, the small number of mothers who smoked 11 or more cigarettes in our study may have made identifying statistically significant differences difficult.

Overall, our results showed that maternal smoking at 6 months of age did not increase the children’s risk of developing BA; however, the maternal smoking group in the presence of paternal smoking significantly increased the risk of developing BA in children. Two prior studies in Japan have shown an association between passive smoking and the risk of developing BA; in these studies, maternal smoking, but not paternal smoking, was associated with the development of BA in offspring; however, both parental smoking significantly increased the risk of BA [12, 23]. Conversely, a birth cohort study of 1,454 children found no association between prenatal, infant, or preschool exposure to passive smoking and BA development [24]. There are two possible explanations for our finding that maternal smoking during infancy did not increase the risk of developing BA in children, which contrasts with the results of previous reports mentioned above. First, compared to these previous studies in Japan, the decreased maternal smoking prevalence during the current study period may have led to a decreased risk of BA in the children. The maternal smoking prevalence was 7.5% in our study, whereas it was much higher at 16.7% or 26.8% in two previous Japanese studies conducted 10 years prior [12, 23]. Second, smoking habits during pregnancy were not investigated in this study; therefore, mothers who stopped smoking after giving birth may have been included in the non-smoking group.

We showed that maternal smoking during infancy did not increase the risk of developing FA in children; however, children in the maternal smoking group in the presence of paternal smoking showed an increased risk of developing FA, although no dose dependence was found. Indeed, one prior study on children reported that exposure to passive smoking during infancy did not increase the risk of developing FA until the age of 16 years [25]. Conversely, one study of 4,089 children exposed to passive smoking during infancy reported that specific immunoglobulin E (IgE) antibodies to the peanut-related allergens were significantly increased at 16 years of age [26]. Another study found that maternal smoking in the perinatal period significantly increased the sensitization to food allergens in children compared to non-exposure; however, paternal smoking did not increase the risk of sensitization [27]. Although the above two reports investigated the risk of sensitization to food antigens due to passive smoking, the presence of food-specific IgE antibodies does not necessarily indicate the development of FA. However, many studies, including ours, did not include a proper diagnosis of FA based on an oral food challenge test; as such, the results may not accurately reflect the risk of developing FA due to passive smoking. Future cohort studies including the diagnosis of FA based on oral food challenge tests are therefore required.

In the present study, we showed that maternal smoking during infancy did not increase the risk of developing AD in children. Conversely, several previous studies have reported that maternal smoking during pregnancy or passive smoking during infancy was associated with an increased risk of developing AD in children [10, 28]. High urinary cotinine levels in mothers during pregnancy are also reported to be associated with the development of AD in children [29]. However, another study reported that exposure to passive smoke during infancy was not associated with the development of AD, which is consistent with our result [11]. Unfortunately, in our study, maternal smoking status during pregnancy was not included in the questionnaire. Conversely, no prior studies have yet investigated the association between paternal smoking and AD risk in children. Interestingly, the present study showed that the maternal smoking effect, modified by paternal smoking during infancy, increases the risk of developing AD in children. Furthermore, an increased number of maternal cigarettes smoked further increased the child’s risk of developing AD.

In the present study, we assessed both additive interactions, by calculating the relative excess risks of maternal and paternal smoking, and multiplicative interactions, by including product terms in the model; however, none of these multiplicative and additive interactions of maternal and paternal smoking showed significance. Based on these results, we divided the groups based on the paternal smoking habits, and evaluated the risk of children developing allergic diseases due to their mothers’ smoking in the present analyses.

This study had some limitations. First, the prevalence of each allergic disease was assessed only based on questionnaires obtained from parents, and it is unclear whether these diseases were accurately diagnosed by pediatric allergists. Second, the same child may develop multiple allergic diseases. This study only discussed the total number of children who developed each allergic disease, and was not a detailed study that considered the comorbidities of each child’s allergic disease. Third, this study did not assess the presence or absence of allergic diseases in the parents; as such, any potential genetic impact of allergic disease in the parents on the children could not be assessed. Fourth, in rare cases, such as children with mothers who were heavy smokers and non-smoking fathers, the number of cases was low. We recognize that this may have introduced problems with statistical power of detection. Finally, this survey did not include smoking habits during pregnancy or before 6 months after birth, or the smoking status of grandparents’ living with them. Exposure of infants to maternal smoking during pregnancy and the neonatal period may influence the risk of developing allergic diseases later in life.

## Conclusions

Overall, this study showed that maternal smoking during infancy increases the infant’s risk of developing future AR/AC, and that maternal smoking in the presence of paternal smoking further increases the infants’ risk of developing various allergic diseases. Although health damage to children caused by passive smoking at home remains an important issue and banning smoking at home and separating smoking areas remain important, there are currently much fewer restrictions on passive smoking in the home than in public places, making children more susceptible to exposure. Efforts by parents to quit smoking at home, or to separate smoking areas, may reduce the children’s risk of developing allergic diseases in the future and improve their quality of life.

## Electronic supplementary material

Below is the link to the electronic supplementary material.

Supplementary Material 1

## Data Availability

The de-identified data analyzed in this study can be requested by contacting the corresponding author.

## Abbreviations

AC: Allergic conjunctivitis
AR: Allergic rhinitis
AD: Atopic dermatitis
BA: Bronchial asthma
FA: Food allergy
CI: Confidence intervals
RR: Risk ratio
