# Supplementary material for: Maternal smoking during infancy increases the risk of allergic diseases in children: a nationwide longitudinal survey in Japan
Source: Allergy Asthma Clin Immunol. 2025 Jan 16;21:4. doi: 10.1186/s13223-025-00952-9 (PMC11740415; doi:10.1186/s13223-025-00952-9)
Supplement: Supplementary file 1 — Supplementary Material 1 [file 13223_2025_952_MOESM1_ESM.docx]

**Supplemental Table**. Baseline characteristics of the parents and infants at 5.5 years of age

|  | Mother | |
| --- | --- | --- |
|  | Smoking  (n = 1,225) | Non-smoking  (n = 25,246) |
| Gender, n (%) | | |
| Male | 616 (50.3) | 13,078 (51.8) |
| Female | 609 (49.7) | 12,168 (48.2) |
| Singleton or multiple birth, n (%) | | |
| Singleton birth | 1,213 (99.0) | 24,783 (98.2) |
| Multiple birth | 12 (1.0) | 463 (1.8) |
| Preterm birth, n (%) | | |
| 22-36 weeks | 63 (5.1) | 1,332 (5.3) |
| 37 weeks or more | 1,162 (94.9) | 23,909 (94.7) |
| Birth weight, n (%) | | |
| <2500 | 1,097 (89.6) | 22,922 (90.8) |
| ≥2500g | 128 (10.4) | 2,319 (9.2) |
| Birth order, n (%) | | |
| First | 450 (36.7) | 12,060 (47.8) |
| Second | 457 (37.3) | 9,588 (38.0) |
| Third or later | 318 (26.0) | 3,598 (14.3) |
| Daycare, n (%) | | |
| Yes | 355 (31.4) | 6,671 (27.8) |
| No | 755 (68.6) | 17,817 (72.8) |
| Mother’s age, n (%) | | |
| <25 | 211 (17.2) | 1,554 (6.2) |
| 25-29 | 325 (26.5) | 6,851 (27.1) |
| 30-34 | 392 (32.0) | 9,907 (39.2) |
| >35 | 297 (24.2) | 6,934 (27.5) |
| Mother’s educational attainment, n (%) | | |
| University or higher | 77 (6.8) | 7,230 (29.6) |
| Junior college | 302 (26.8) | 10,463 (42.8) |
| High school | 531 (47.1) | 6,008 (24.6) |
| Junior high school or other | 218 (19.3) | 743 (3.0) |
| Father’s educational attainment, n (%) | | |
| University or higher | 165 (15.6) | 11,563 (47.9) |
| Junior college | 173 (16.4) | 4,487 (18.6) |
| High school | 508 (48.1) | 6,866 (28.4) |
| Junior high school or other | 210 (19.9) | 1,249 (5.2) |
| Residential area, n (%) | | |
| Wards | 332 (27.1) | 7,339 (29.1) |
| City | 768 (62.7) | 15,950 (63.2) |
| Town or villages | 125 (10.2) | 1,957 (7.8) |
| Pet ownership (cat and/or dog), n (%) | | |
| Yes | 258 (22.9) | 2,890 (12.1) |
| No | 867 (77.1) | 20,920 (87.9) |
